# Culture, behavior and health

**DOI:** 10.1093/emph/eoz036

**Published:** 2019-12-16

**Authors:** Margarita Hernandez, James K. Gibb

**Affiliations:** 1 Department of Anthropology, Pennsylvania State University, University Park, State College, PA 16802, USA; 2 Department of Anthropology, University of Toronto, Toronto, Ontario, Canada

**Keywords:** anthropology, culture, health, behavior

## DEFINITION AND BACKGROUND

Cultural behaviors have important implications for human health. Culture, a socially transmitted system of shared knowledge, beliefs and/or practices that varies across groups, and individuals within those groups, has been a critical mode of adaptation throughout the history of our species [1]. Socioeconomic status, gender, religion and moral values all play into how individuals experience, conceptualize and react to their world, and therefore general understandings of cultural groups are insufficient for grasping a patient’s unique experience with health and illnesses [2, 3]. Additionally, structural inequalities and political economy play a critical, and often overlooked, role in health and disease [4]. Understanding how behaviors are rooted in an individual’s unique cultural experience and as a response to social pressures can better equip medical professionals with the context, skills and empathy necessary for holistic care [2].

Healthcare providers can improve individual outcomes by thoroughly factoring in life experiences as part of understanding an individual’s health and treating their illnesses. The use of a ‘mini-ethnography’ can help healthcare providers understand how identity, interpretation of illness and the moral values of patients factor into building a trusting relationship that considers the patient’s life experiences into treatment plans [3]. Table 1 summarizes this approach.

**Table 1.** Kleinman and Benson’s approach to conducting a ‘mini-ethnography’ with every patient in order to best incorporate a patient’s culture into treatment plans [3]

Steps for performing a mini-ethnography	Description
**Step 1:** How does ethnicity factor into your patient’s identity?	Not all individual’s identify with their ethnicity. Ask your patient how they identify with theirs and the importance their ethnicity plays in their life.
**Step 2:** What is at stake for your patient and their loved ones?	Illnesses can jeopardize aspects of patients’ lives in ways that may not be immediately visible. Ask your patient what is at stake in having this illness.
**Step 3:** How does your patient conceptualize their illness?	Individuals may conceptualize their illness differently than healthcare providers. Ask your patient what they call their illness, what they believe the cause of their illness may be, what they believe potential treatments are, and what they fear most about treatment.
**Step 4:** What social stresses is your patient experiencing because of their illness?	Ask your patient what additional stressors they may be experiencing because of their illness. These can include financial, familial and professional stressors that may impact their treatment plan.
**Step 5:** How does the clinical setting influence your relationship with your patient?	Determine and acknowledge the extent to which the clinical setting may influence your patient. How does the culture of biomedicine influence your patient’s ability to seek and receive treatment for their illness?
**Step 6:** Is this intervention appropriate for your patient?	Determine what clinical interventions would be appropriate for your patient, not necessarily for the illness. This should factor in the information you’ve gathered from the previous steps.

## EXAMPLES IN HUMAN BIOLOGY AND PUBLIC HEALTH

In rural Bolivia, children of mothers with higher indices of local ecological knowledge (LEK) had reduced inflammation, taller height, and less hookworm infections than children of mothers with lower indices of LEK [5, 6].

The Acholi people of Uganda have several cultural models for understanding and responding to disease outbreaks that were employed during the 2000 Ebola outbreak [7]. Acholi cultural practices related to *gemo*, or an epidemic outbreak, limit the spread of infectious diseases that may have occurred through traditional funerary practices, such as the washing and touching of deceased bodies [7].

Both examples highlight a need for understanding Indigenous knowledge systems as they relate to health and in responding to disease.

## EXAMPLE IN CLINICAL MEDICINE

Understanding how social pressures, such as racism and discrimination, manifest biologically is critical in understanding how cultural behavior relates to health. In a sample of diverse pregnant women in New Zealand, those that experienced ethnic discrimination had high cortisol levels and their infants higher cortisol reactivity, suggesting a transgenerational effect of discrimination [8]. 
